# Supplementary material for: Combined modelling of mRNA decay dynamics and single-molecule imaging in the Drosophila embryo uncovers a role for P-bodies in 5′ to 3′ degradation
Source: PLoS Biol. 2023 Jan 17;21(1):e3001956. doi: 10.1371/journal.pbio.3001956 (PMC9882958; doi:10.1371/journal.pbio.3001956)
Supplement: S1 Supplementary methods — (PDF) [file pbio.3001956.s006.pdf]

Supplementary computational methods for "Combined modelling of mRNA decay dynamics and single molecule imaging in the *Drosophila* embryo uncovers a role for P-bodies in $5'$ to $3'$ degradation"

This supplementary file provides detailed information on the filtering scheme, the transcriptional regulation model with Gaussian processes used in this paper, and inference methods, including Markov Chain Monte Carlo sampling.

Filtering of differentially expressed genes

We used the approach introduced in [1] to filter out the genes which do not exhibit time-dependent dynamics. We used two Gaussian processes regressions of the form

$$y = f(t) + \epsilon, \tag{1}$$

where $f(\cdot)$ is a Gaussian process and ϵ is Gaussian noise with zero mean and variance σ^2. We fit two models to the data: 1) a Gaussian processes regression with GP defined by a noise kernel, i.e. $f(x) \sim GP(0, K_{noise})$; 2) a Gaussian process regression with GP defined by an RBF kernel, i.e. $f(x) \sim GP(0, K_{RBF})$. We kept only the genes which exhibited the dynamics when comparing the null model with no time-dependence (noise kernel) and the model with time-dependence (RBF kernel).

Model formulation through differential equations

The level of mRNA can be related to the level of pre-mRNA and depends both on the splicing efficiency and degradation rate. The dynamics of mRNA production is modelled by an ordinary differential equation (ODE). The intronic bases of the pre-mRNA ($p(t)$) and the exonic bases of the mature mRNA ($m(t)$) are assumed to be transcribed at the same time-varying gene-specific rate $r(t)$

$$\frac{dp}{dt} = r(t) - Sp(t) \ , \tag{2}$$

$$\frac{dm}{dt} = r(t) - Dm(t) \ , \tag{3}$$

where S is the splicing rate and D is the mRNA degradation rate. In *Drosophila*, splicing is typically fast (median half-life 2 minutes) and therefore we take the large S limit to get $r(t) = Sp(t)$. Consequently, the above equations can be simplified into an ODE relating the transcription rate of mRNA to the level of pre-mRNA

$$\frac{dm}{dt} = Sp(t) - Dm(t) \ . \tag{4}$$

The solution to Equation (4) is

$$m(t) = Se^{-Dt} \int_0^t p(u) e^{Du} du \ . \tag{5}$$

In [2] the transcription rate is modeled non-parametrically as a Gaussian process (GP) based on earlier work modelling TF regulatory activities [3]. Following this approach we say that $p(t) \sim GP(0, \Sigma)$, where Σ is defined by a squared exponential or RBF kernel. Note, that in Equation (5) the dependence of $m(t)$ on GP is linear since integration is a linear operation. It follows that $m(\cdot)$ is also a Gaussian process, albeit with a different kernel. For more detailed derivations of this kernel we refer to [3] and [2], while we discuss the exact kernel function and implementation of the GP regression following described ODEs in the next section.

Observational model with noise and GPflow implementation

We assume an observational model with noise, and thus in practice, we observe $y^m(t)$ and $y^p(t)$ defined as

$$y^m(t) = m(t) + \epsilon^m(t), \tag{6}$$

$$y^p(t) = p(t) + \epsilon^p(t), \tag{7}$$

where the noise terms $\epsilon^m(t)$ and $\epsilon^p(t)$ are assumed to be Gaussian with mean zero and different variances. We assume the following RBF kernel for $p(t)$:

$$k_p(t, t') = V \exp\left(-\frac{(t-t')^2}{l^2}\right). \tag{8}$$

The kernel for $m(\cdot)$ and cross-covariance between $m(\cdot)$ and $p(\cdot)$ are derived in [3]. The kernel for $m(\cdot)$ reads

$$k_m(t, t') = VS^2 \frac{\sqrt{\pi} l}{2} \left[h(t', t) + h(t, t')\right] \tag{9}$$

where

$$h(t', t) = \frac{\exp(\gamma)^2}{2D} \left\{ \exp[-D(t'-t)] \left[erf\left(\frac{t'-t}{l} - \gamma\right) + erf\left(\frac{t}{l} + \gamma\right)\right] \right. \\ \left. - \exp[-(Dt' + D)] \left[erf\left(\frac{t'}{l} - \gamma\right) + erf(\gamma)\right] \right., \tag{10}$$

where $\gamma = \frac{Dl}{2}$.
The cross-covariance between $m(\cdot)$ and $p(\cdot)$ is

$$k_{mp}(t', t) = V \frac{\sqrt{\pi} l S}{2} \exp(\gamma)^2 \exp\left[-D(t'-t)\right] \left[erf(\frac{t'-t}{l} - \gamma) + erf(\frac{t}{l} + \gamma)\right]. \tag{11}$$

We write the complete GP regression model by combining the data for mRNA and pre-mRNA into a joint vector $\boldsymbol{y} = (\boldsymbol{y^m}, \boldsymbol{y^p}) \sim GP(0, K_y + K_{noise})$, where joint covariance matrices for ODE-like dynamics and noise terms are given by

$$K_y = \begin{pmatrix} K_m & K_{mp} \\ K_{pm} & K_p \end{pmatrix}, \quad K_{noise} = \begin{pmatrix} \sigma_m^2 I & 0 \\ 0 & \sigma_p^2 I \end{pmatrix}. \tag{12}$$

We use GPflow library [4] for implementation, the block kernel described in Equation (12) is coded manually, but the routines for the optimization of the parameters are standard.

Marginal likelihood

The marginal likelihood of the model is Gaussian and is used to optimize model parameters. For numerical stability, log-marginal likelihood is used:

$$\log p(y|t,\theta) = -\frac{1}{2}y^T K_y^{-1} y - \frac{1}{2}\log|K| - \frac{N}{2}\log 2\pi, \tag{13}$$

Maximum a posteriori (MAP) parameter estimates are obtained by maximising this quantity added to the logged hyper-parameter priors (defined in the next section).

Markov Chain Monte Carlo (MCMC) sampling from the posterior distribution

We use the Metropolis-adjusted Langevin algorithm (MALA) sampling scheme for fully Bayesian inference of the genes of interest. Using MALA, we obtain posterior samples to quantify uncertainty about the parameters of the model. MALA is a gradient-based MCMC algorithm that exploits Langevin dynamics for the proposal mechanism. Generally, this allows obtaining a higher acceptance rate while making large proposal steps and exploring parameter space sufficiently well.
We use the implementation of MALA from the Tensorflow probability library while the model itself is implemented in the GPflow library. For the sampling, all of the parameters are transformed to the unconstrained space using logistic transformation.
The following prior distributions were chosen for the model parameters: $S \sim Gamma(2,2)$, $D \sim Gamma(2,2)$, $\sigma_m^2 \sim Gamma(\alpha_m, \beta_m)$ and $\sigma_p^2 \sim Gamma(\alpha_p, \beta_p)$, where hyperparameters α_m, β_m, α_p, β_p depend on the empirical mean and standard deviation.
We run MALA with a different number of step sizes $[10^{-5}, 10^{-4}, 10^{-3}, 0.003, 0.005, 0.01, 0.03, 0.05, 0.07, 0.1, 0.15, 0.2, 0.25, 0.3, 0.35, 0.5, 1]$ and pick the one which results in higher acceptance rate/-better mixing of the posterior distribution.

Illustrative examples

This section uses two sets of examples to demonstrate the model's performance. In the first set of examples, we simulated five data sets with different degradation rates – 0.003, 0.008, 0.01, 0.02, 0.05 – which correspond to 231.0, 86.6, 69.3, 34.6, and 13.8 min half-lives, respectively. Figures S1**a**, **c**, **e**, **g**, **i** illustrate simulated data and fitted models and Figures S1 **b**, **d**, **f**, **h**, **j** illustrate corresponding posterior estimates for the degradation parameter obtained with MCMC and with maximum a posteriori estimation. For all of the settings, the true degradation

parameter is inferred accurately using both the maximum a posteriori approach and MCMC sampling.
In the second set of examples, we focus only on small degradation rates – 0.008, 0.003, 0.001, 0.0005 – which correspond to 86.6, 231.0, 693.1, and 1386.2min half-lives, respectively. We investigate the limit of the temporal scale of the half-lives, which is identifiable by the model. Figures S2 **a**, **b** , **c**, **d** illustrate model fits for the small degradation examples and the estimates of the degradation obtained with maximum a posteriori estimation. In Figures S2**a** and **b**, the degradation rates, 0.008 and 0.003, are estimated well and are still identifiable. However, in Figures S2**c** and **d**, the model cannot distinguish between degradation parameters 0.001 and 0.0005 (693.1min and 1386.2min half-lives respectively). When the degradation is zero, the half-life is practically indistinguishable from infinity, and thus, for small degradations (large half-lives), the parameter becomes non-identifiable.
In both sets of examples, other model parameters for data generation were fixed to $S = 0.3$, $V = 69.0$, $l = 33.0$, $\sigma_m^2 = 50.5$, $\sigma_p^2 = 3.5$.

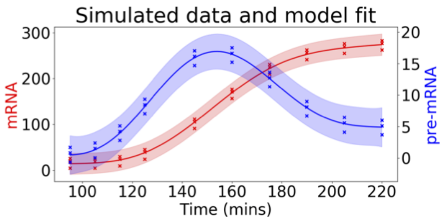

a D=0.003, half-life=231.0 min

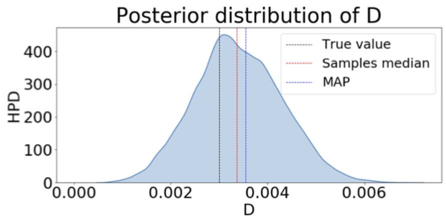

b D_{map} = 0.0035, D_{med} = 0.0033

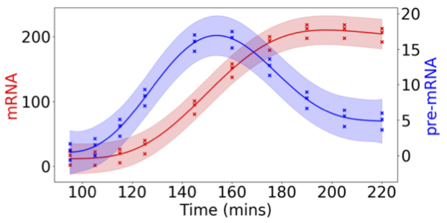

c D=0.008, half-life=86.6 min

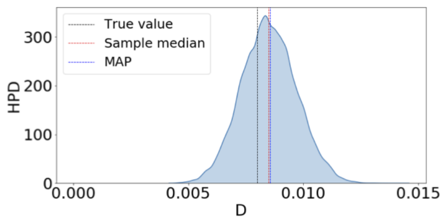

d D_{map} = 0.0085 , D_{med} = 0.0084

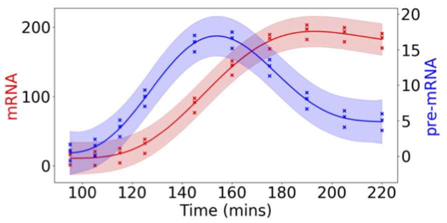

e D=0.01 half-life=69.3 min

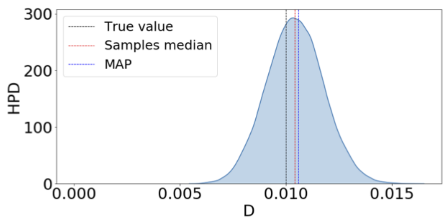

f D_{map} = 0.01 , D_{med} = 0.01

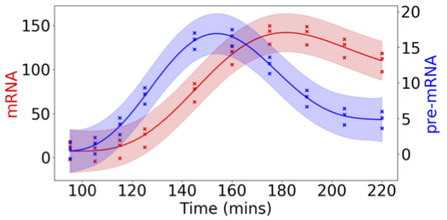

g D=0.02, half-life=34.6 min

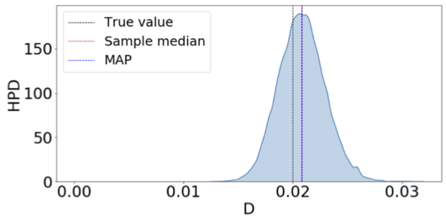

h D_{map} = 0.02 , D_{med} = 0.02

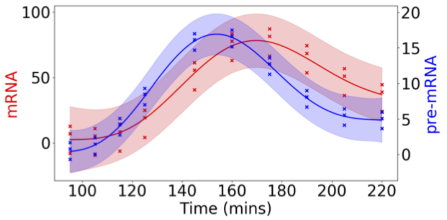

i D=0.05, half-life=13.8 min

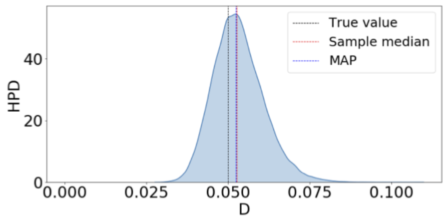

j D_{map} = 0.052 , D_{med} = 0.052

Figure S1: *Left column*: simulated data and model fits of the transcriptional regulation model. *Right column*: posterior density estimates for the degradation parameters, where D_{map} indicates maximum a posteriori estimate and D_{med} indicates MCMC sample median.

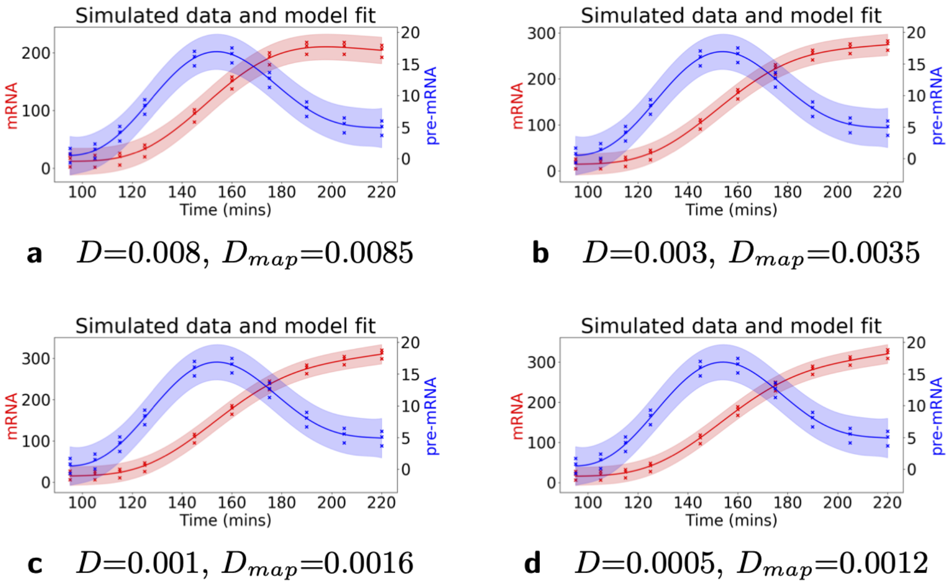

Figure S2: Examples of model fits and maximum a posteriori estimates (D_{map}) for small D values/long half-lives. **a** $D = 0.008$ corresponds to 86.6min half-life. **b** $D = 0.003$ corresponds to 231.0min half-life. **c** $D = 0.001$ corresponds to 693.1min half-life. **d** $D = 0.0005$ corresponds to 1386.2min half-life. When D approaches zero, it becomes unidentifiable.

References

[1] A. A. Kalaitzis and N. D. Lawrence, "A simple approach to ranking differentially expressed gene expression time courses through Gaussian process regression," *BMC bioinformatics*, vol. 12, no. 1, pp. 1–13, 2011.

[2] A. Honkela, J. Peltonen, H. Topa, I. Charapitsa, F. Matarese, K. Grote, H. G. Stunnenberg, G. Reid, N. D. Lawrence, and M. Rattray, "Genome-wide modeling of transcription kinetics reveals patterns of RNA production delays," *Proceedings of the National Academy of Sciences*, vol. 112, no. 42, pp. 13115–13120, 2015.

[3] N. D. Lawrence, G. Sanguinetti, and M. Rattray, "Modelling transcriptional regulation using Gaussian processes," *Advances in Neural Information Processing Systems*, vol. 19, p. 785, 2007.

[4] A. G. d. G. Matthews, M. Van Der Wilk, T. Nickson, K. Fujii, A. Boukouvalas, P. León-Villagrá, Z. Ghahramani, and J. Hensman, "GPflow: A Gaussian Process Library using TensorFlow.," *J. Mach. Learn. Res.*, vol. 18, no. 40, pp. 1–6, 2017.
