# Supplementary figures and images for: Combined modelling of mRNA decay dynamics and single-molecule imaging in the Drosophila embryo uncovers a role for P-bodies in 5′ to 3′ degradation
Source: PLoS Biol. 2023 Jan 17;21(1):e3001956. doi: 10.1371/journal.pbio.3001956 (PMC9882958; doi:10.1371/journal.pbio.3001956)

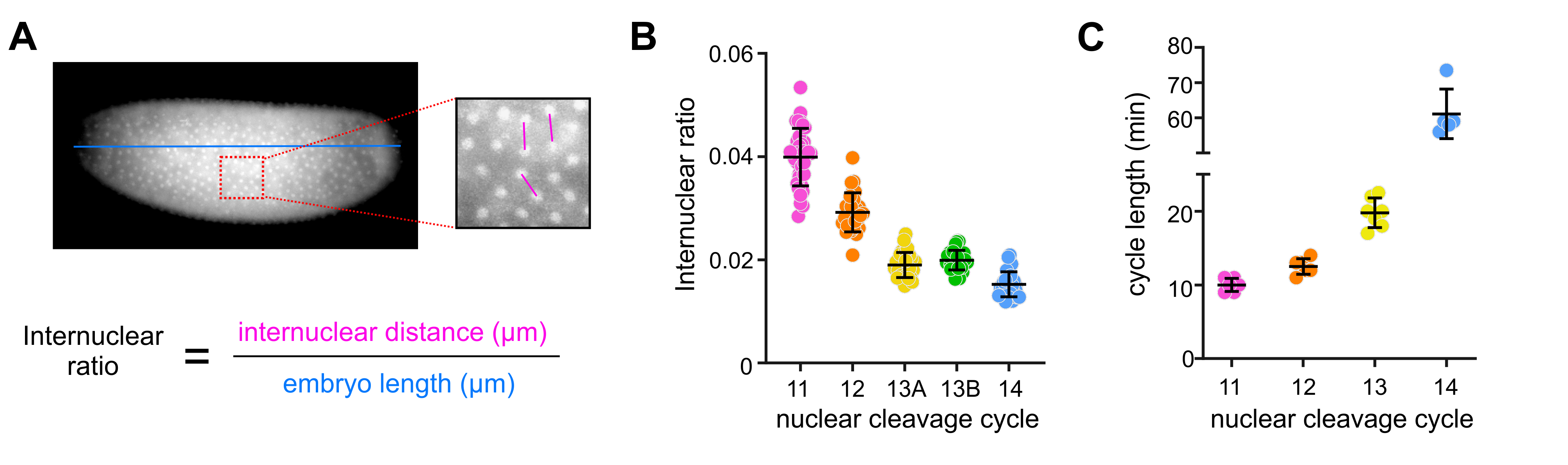

Supplement: S1 Fig — (A) Images of embryos were captured immediately prior to collection, and the internuclear distance and embryo length were measured for each to give an internuclear ratio. (B) The internuclear ratio at nuclear cleavage cycles (nc) can be used to accurately stage embryos. (C) Timing of each nc at 20°C used in experiments. Underlying data can be found in S1 Data. (TIF) [file pbio.3001956.s007.tif]

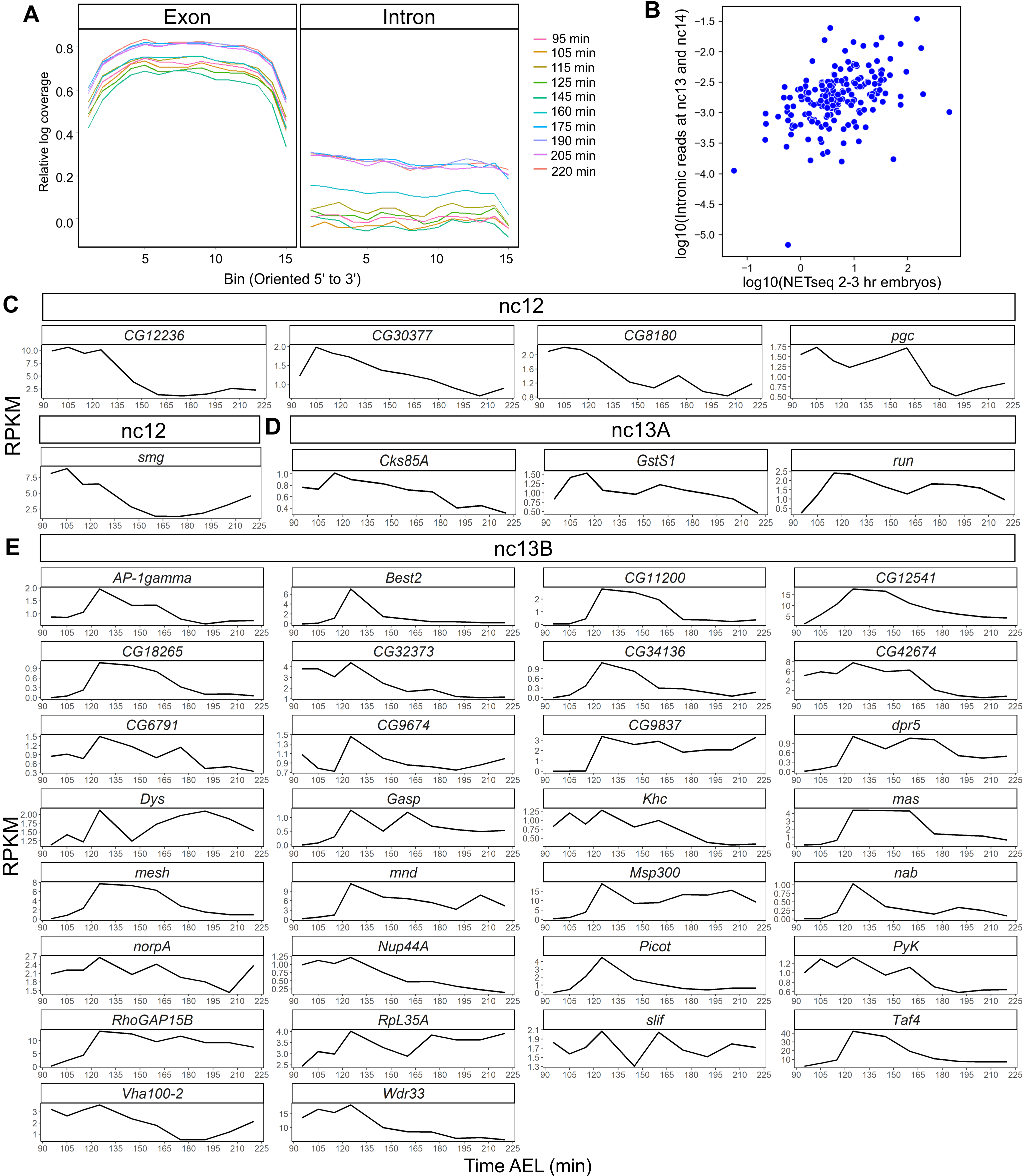

Supplement: S2 Fig — (A) Binned read coverage across exons and introns for 1 replicate for each of the time points within the time series. (B) Scatterplot of our RNA-seq reads versus NET-seq read counts showing a relationship (Spearman’s Rank correlation ρ = 0.46, p = 5.1 × 10^−11^). Intronic read counts (RPKM) of genes that show early transcription in our dataset, grouped depending on peak expression at (C) nc12 (105 min), (D) nc13A (115 min), or (E) nc13B (125 min), based on timings at 20°C. Underlying data can be found in S1 Data. (TIF) [file pbio.3001956.s008.tif]

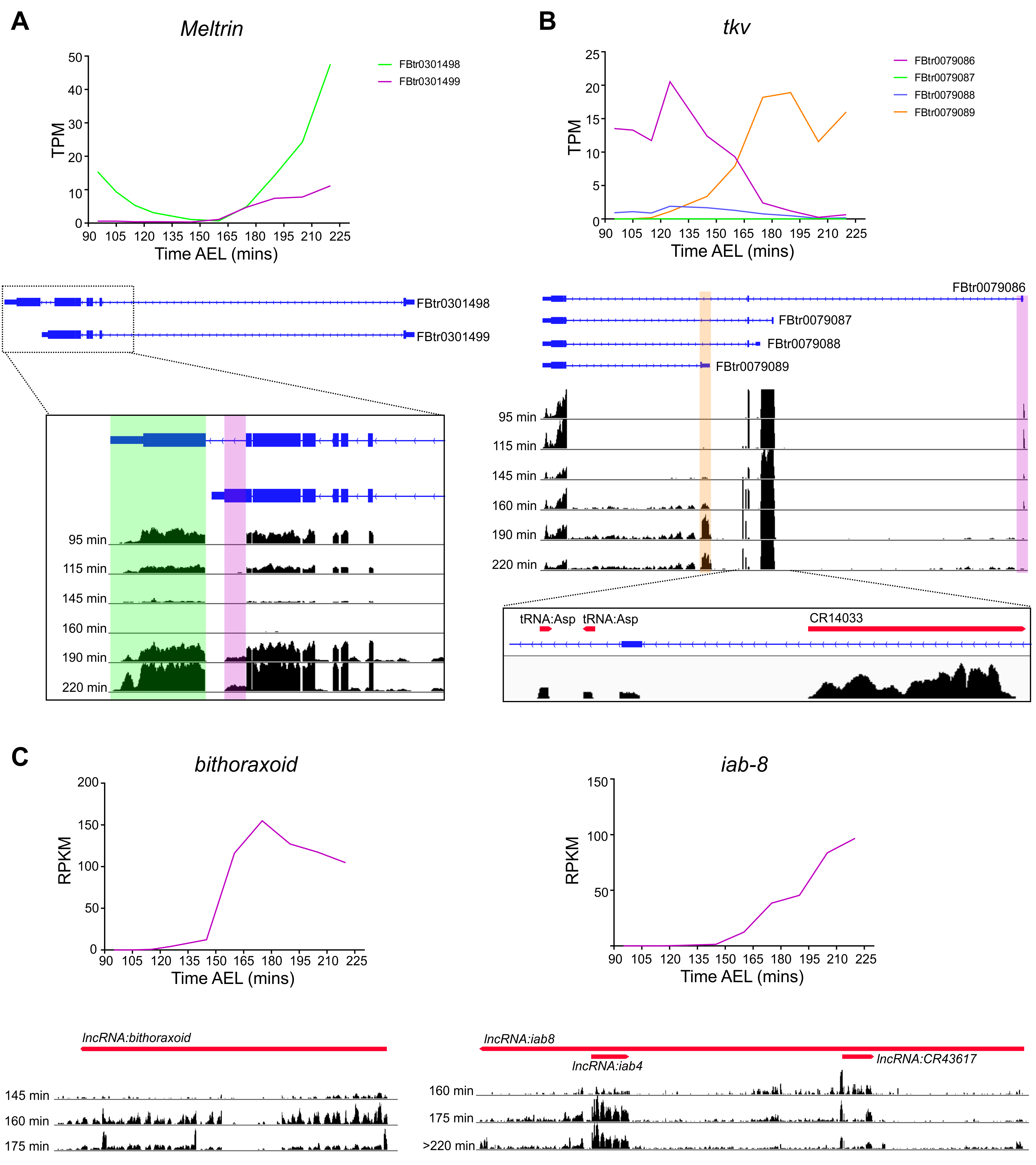

Supplement: S3 Fig — (A) Quantification of the 2 different transcripts (TPM) of the Meltrin gene. The FBtr0301499 isoform (purple) is not detected in embryos <160 min AEL but is detected at later time points in addition to the FBtr0301498 (green) transcript. Mapped reads are shown below with a region highlighted in purple depicting the increase in the alternative transcript at time point 190 and 220 min. (B) As in (A) but for the gene tkv. Transcription of the FBtr0079086 (purple) and FBtr0079089 (orange) isoforms switches during the time course of development, as highlighted on the mapped reads below. Expression of noncoding RNAs, including 2 copies of tRNA-Asp and a pseudogene (CR14033), overlap the tkv locus as shown in the expanded region below from time point 220 min. (C) Expression of the noncoding RNAs bithoraxoid and iab-8, iab-8 also has 2 overlapping ncRNAs. Gene-level counts (RPKM) show dynamic expression across the time course of these 2 noncoding RNAs. Gene regions for the ncRNAs (red) are shown with the genome browser tracks below. Underlying data can be found in S1 Data. (TIF) [file pbio.3001956.s009.tif]

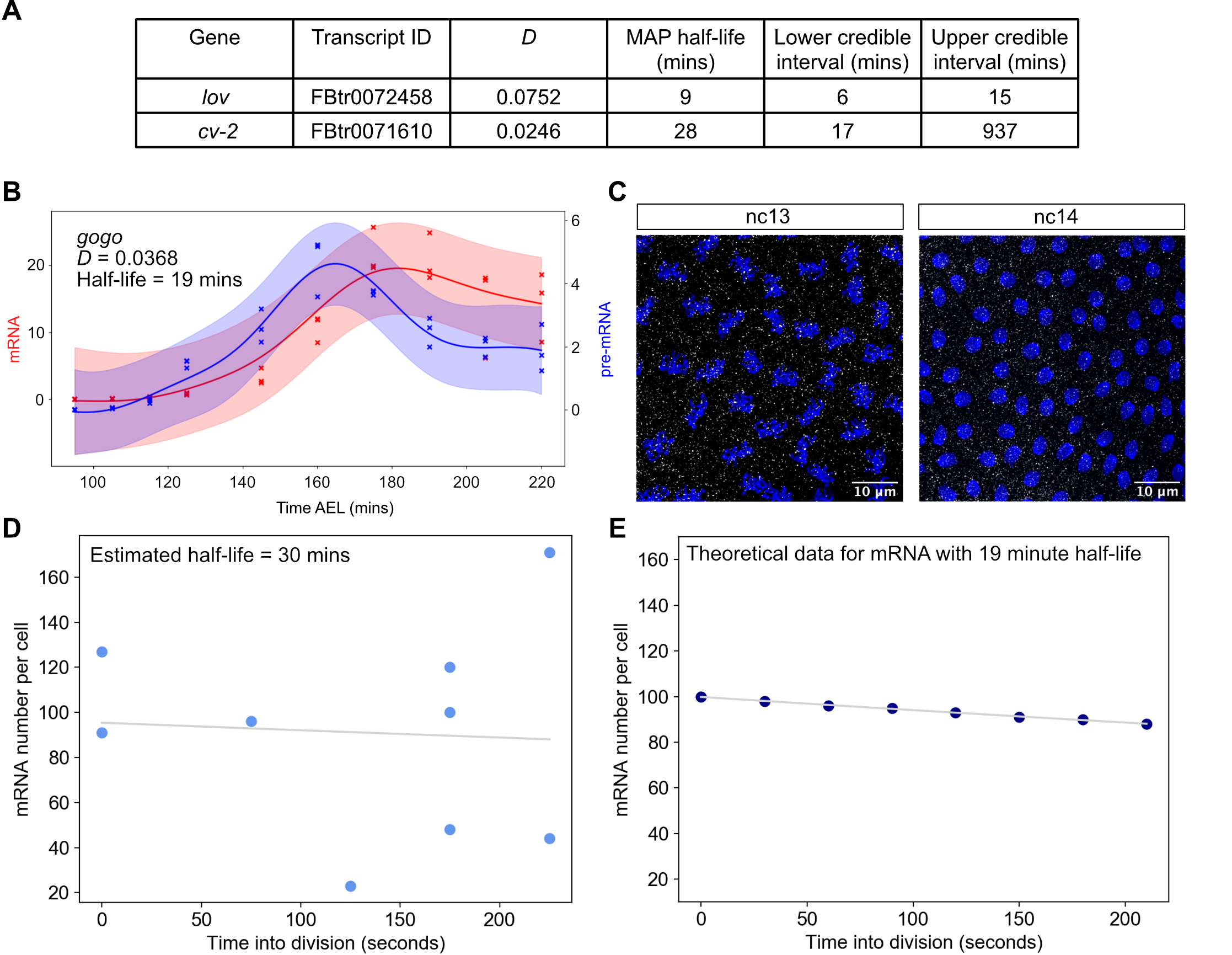

Supplement: S4 Fig — (A) Parameter estimates for degradation rates and credible regions of short and long half-life mRNAs shown in Fig 2D. (B) RNA-seq data for gogo fitted with the Gaussian process model. A half-life of 19 min is inferred for this mRNA. (C) Confocal images of pre- and post-division during the 13th and 14th nuclear cycles showing nuclei stained with DAPI (blue) and single mRNAs (white). (D) Data for the number of mRNAs per cell for embryos at various time points during the cell division (embryo timings are based on referencing live imaging movies, see Methods), fitted with an exponential function from which a half-life of 30 min was calculated. The data has a low signal to noise ratio of 0.0013, meaning that the reduction in transcript numbers over time due to degradation (signal) is much smaller than the natural embryo to embryo variation in transcript numbers (noise). (E) Theoretical data demonstrating the reduction in transcript numbers that would be expected for an mRNA with a 19 min half-life over a 210-s time frame, which is a reduction of approximately 10%. Underlying data can be found in S1 Data. (TIFF) [file pbio.3001956.s010.tiff]

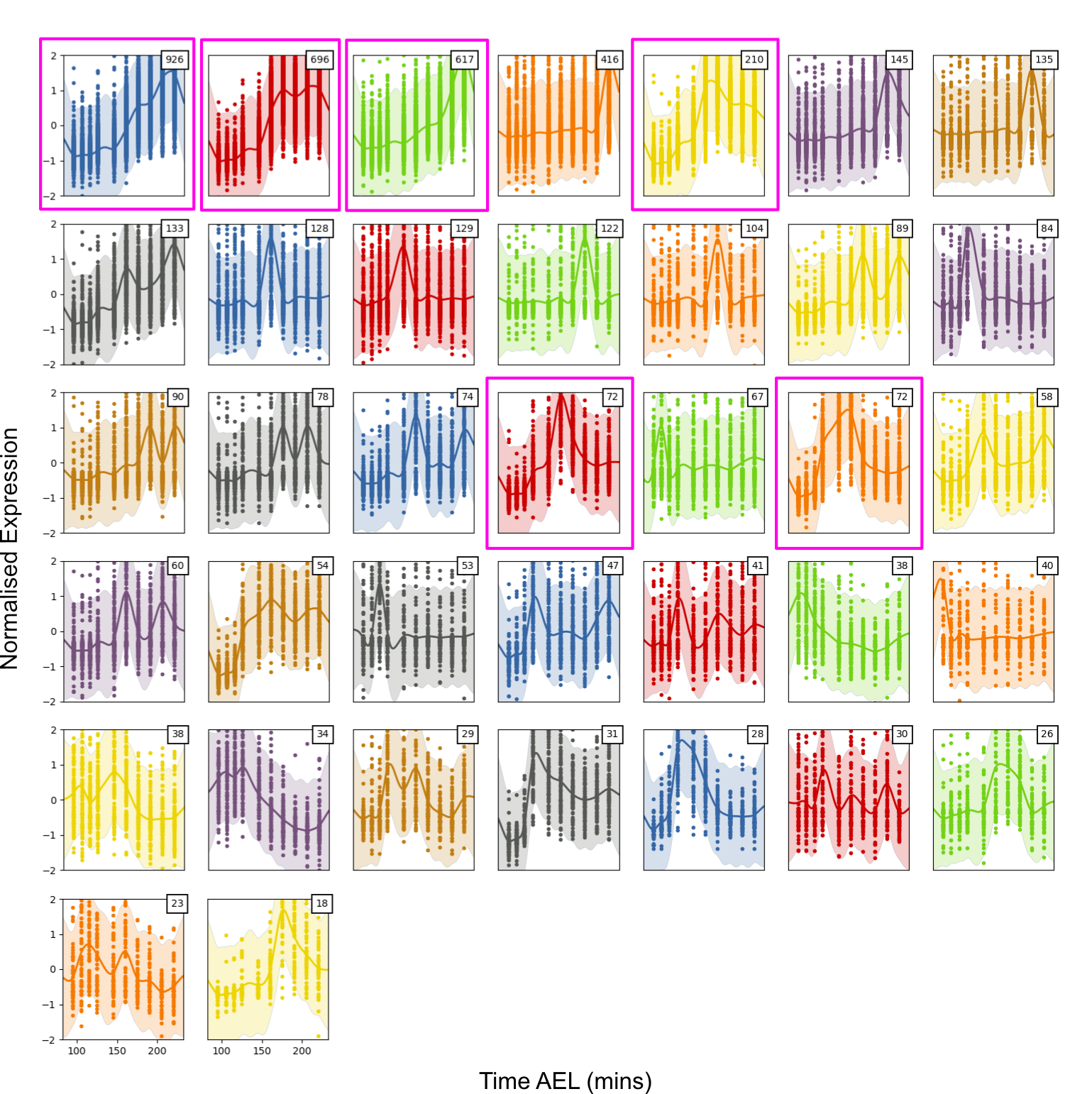

Supplement: S5 Fig — Data for all pre-mRNAs in the cluster are shown with shaded credible regions and inferred function as a solid line. The number of pre-mRNAs in each cluster is shown in the top right corner of each plot and graphs are arbitrarily coloured. Clusters that show interesting dynamics and contain high numbers of pre-mRNAs (1, 2, 3, 5, 18, 20) are highlighted and also displayed in Fig 3Ai. Underlying data can be found in S1 and S4 Tables. (TIFF) [file pbio.3001956.s011.tiff]

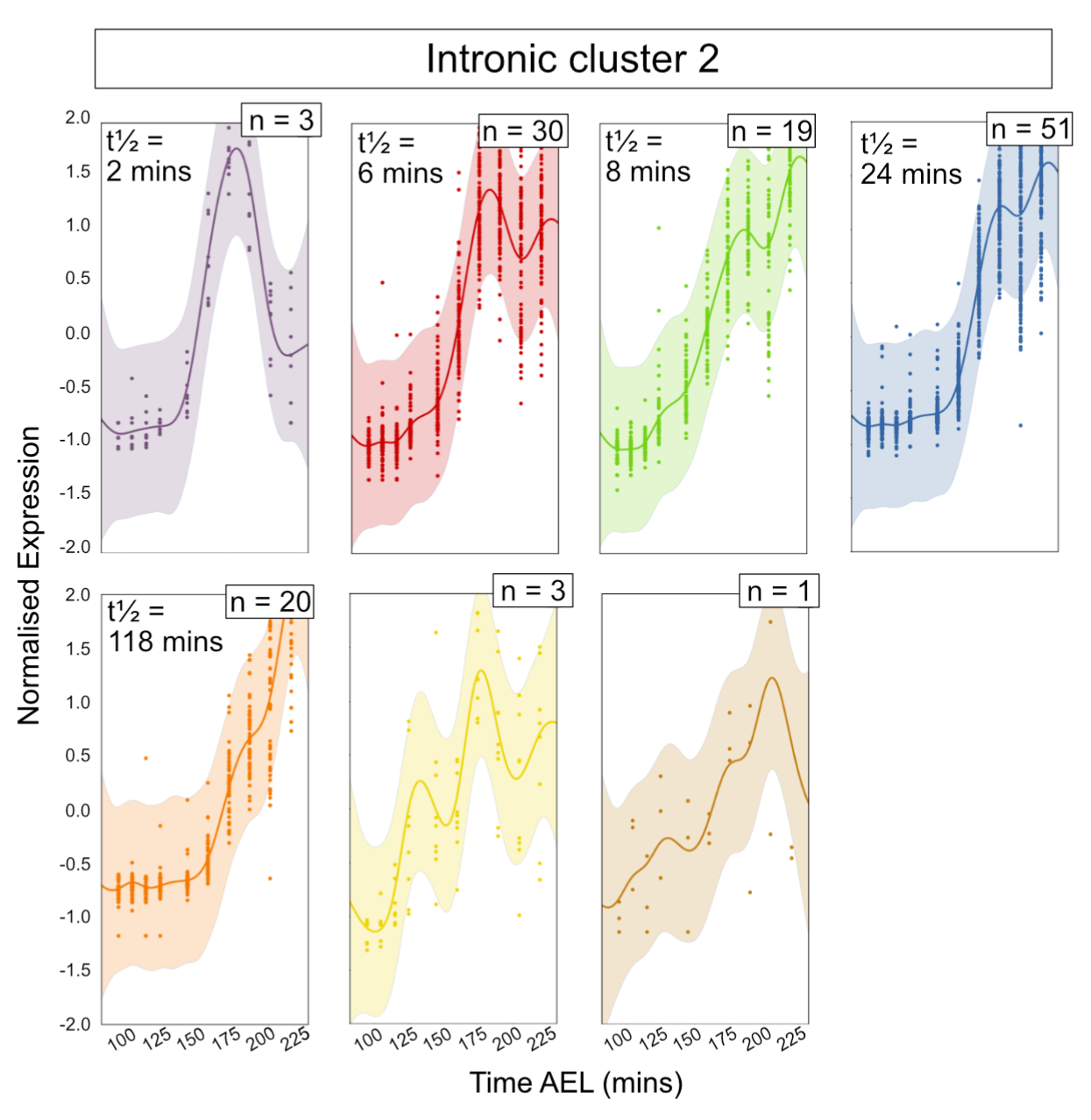

Supplement: S6 Fig — Data for all mRNAs in intronic cluster 2 are shown with shaded credible regions and inferred function as a solid line. The number of mRNAs in each cluster is shown in the top right corner of each plot and graphs are arbitrarily coloured. The mean half-lives of the transcripts in the cluster are shown where the cluster has >2 transcripts with estimated half-lives. Underlying data can be found in S1 and S4 Tables. (TIFF) [file pbio.3001956.s012.tiff]

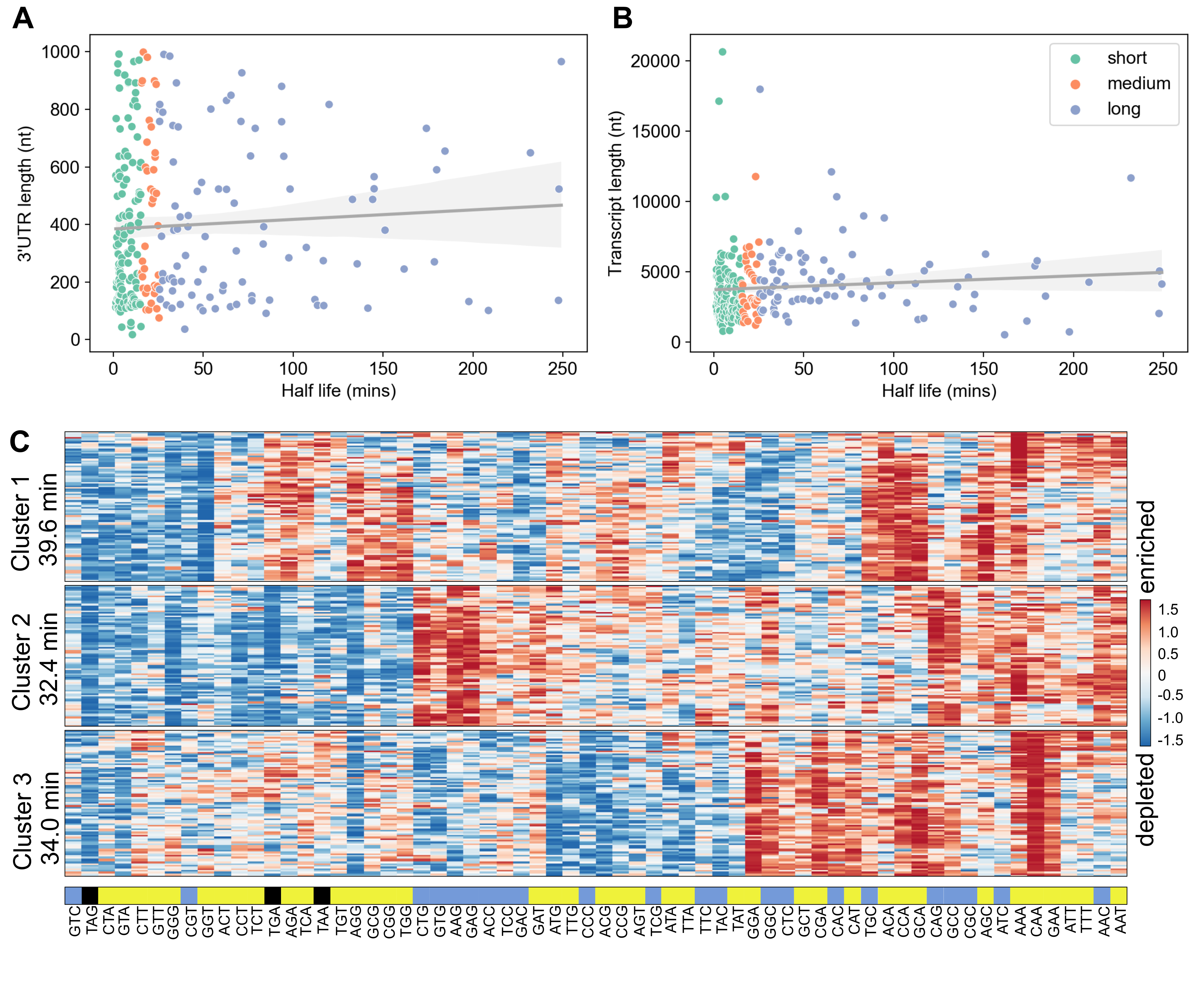

Supplement: S7 Fig — (A) Graph showing the correlation between mRNA half-life and 3′ UTR length. Data fit with linear regression model, Pearson’s r = 0.06, p = 0.34. (B) Correlation between mRNA length and half-life. Transcripts are coloured by half-life category short (green), medium (orange), or long (blue). Data fit with linear regression model, Pearson’s r = 0.09, p = 0.13. (C) Transcripts were clustered based on similar codon usage using K-means and the level of enrichment of each codon within the transcript is shown in the heatmap. The average half-life of each cluster is shown to the left. Codons are coloured by whether they are optimal (blue) vs. non-optimal (yellow) with stop codons shown in black. Underlying data can be found in S1 Data. (TIF) [file pbio.3001956.s013.tif]

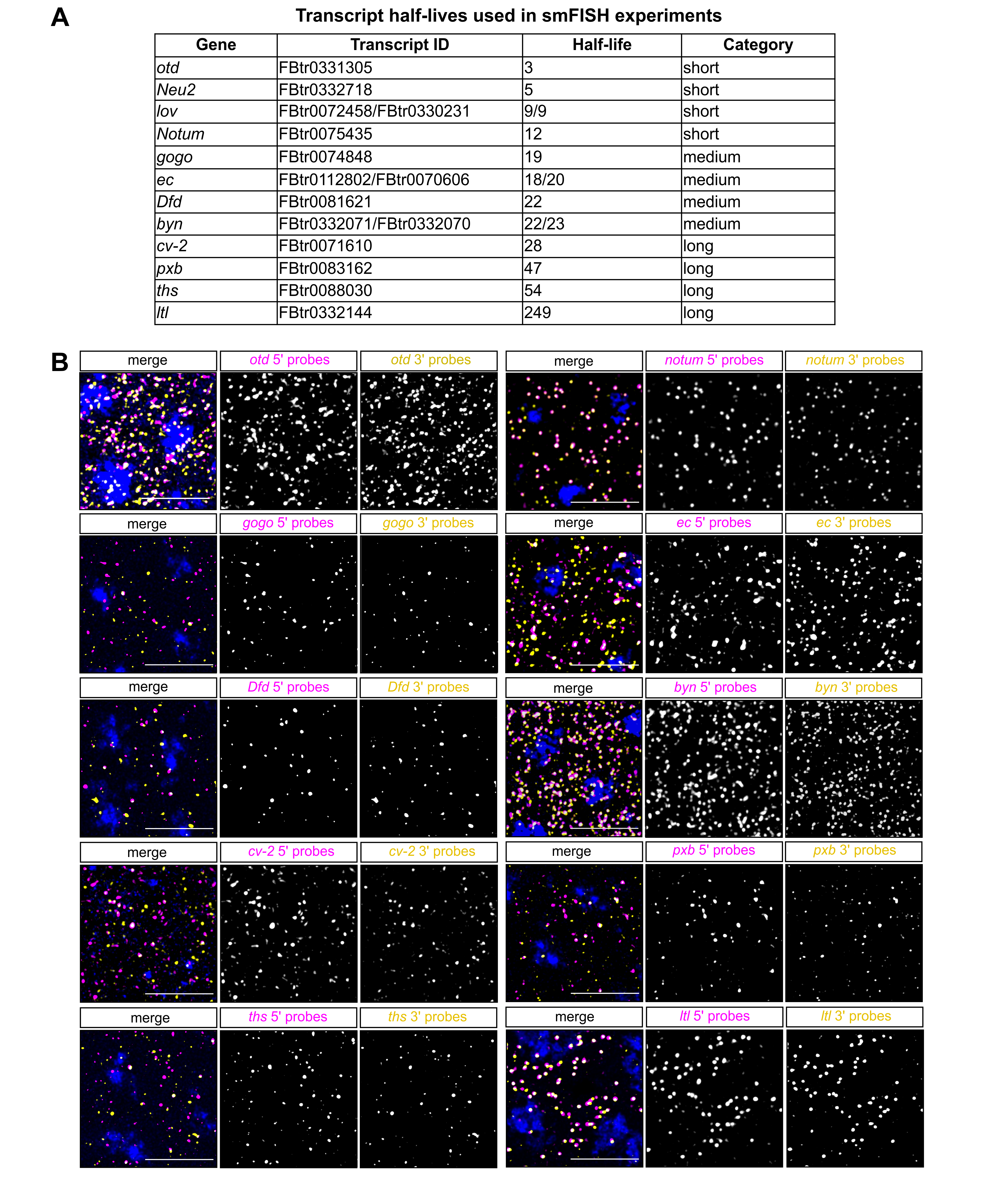

Supplement: S8 Fig — (A) A table of the transcripts used in smFISH experiments. (B) Confocal images of fixed embryos showing smFISH detection of the 5′ (magenta) and 3′ (yellow) ends of the mRNAs used in the compaction experiments quantitated in Fig 4F. Scale bars: 5 μm. (TIF) [file pbio.3001956.s014.tif]

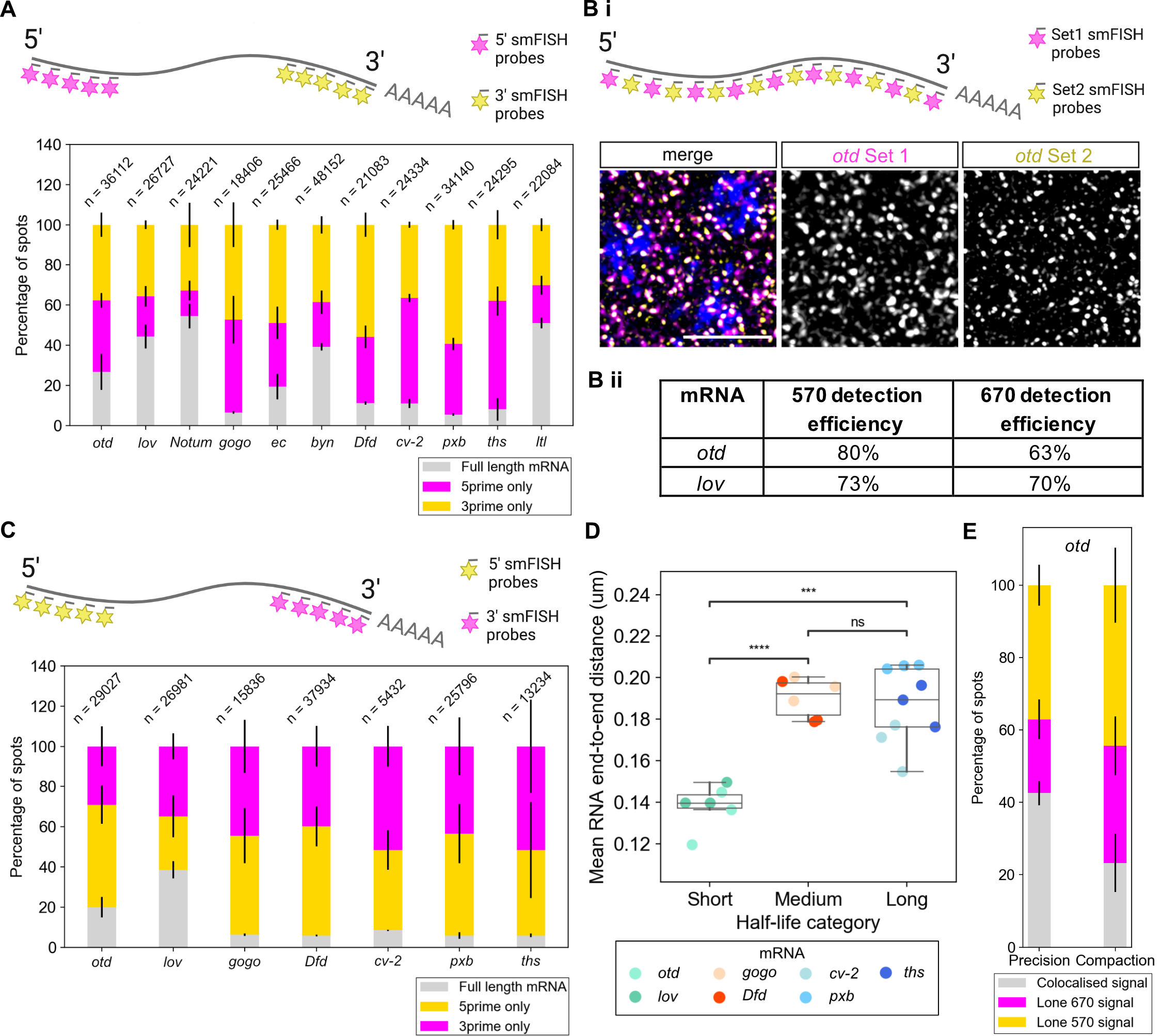

Supplement: S9 Fig — (A) Proportions of 5′ (670) and 3′ (570) ends and whole mRNAs detected in smFISH experiments for all the compaction forward data described in Fig 4F. Numbers represent total mRNAs in the image (whole, 5′ only and 3′ only). Schematic illustrates the forward staining scheme. (Bi) Schematic shows full-length otd mRNAs detected using probes with alternating labels used as a precision control. Confocal images of fixed embryos stained with alternating smFISH probes. All images are maximum projections of 7 Z slices. Scale bars: 5 μm. (Bii) Table of detection efficiencies for the 2 mRNAs used in precision control experiments. (C) Proportions of 5′ (570) and 3′ (670) ends and whole mRNAs detected in smFISH experiments for reverse data (with switched fluorophores). Numbers represent total mRNAs in the image as in A. (D) Graph shows the end-to-end distances of mRNAs with different stabilities in the reverse fluorophore experiment to Fig 4F, data are shown in boxplots for each half-life category with n = 3 embryos for each transcript. (E) Proportion of otd signals detected in only the 570 or 670 channel, or colocalised signal in the precision or both compaction experiments. Underlying data can be found in S1 Data. (TIFF) [file pbio.3001956.s015.tiff]

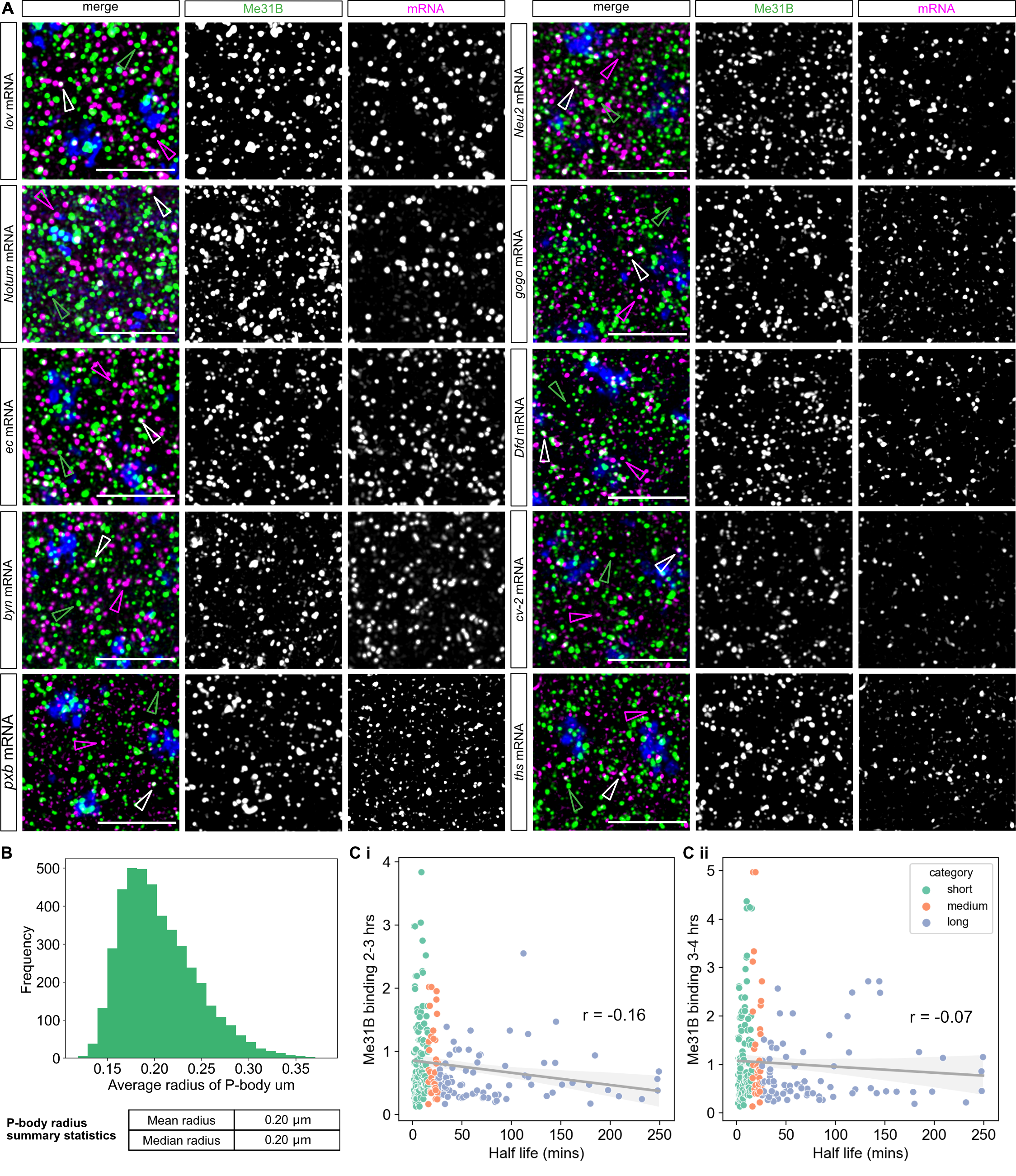

Supplement: S10 Fig — (A) Confocal images of early nc14 Me31B-GFP embryos showing smFISH staining of the indicated test mRNAs. DAPI marks the nuclei (blue), mRNAs are shown in magenta (magenta arrowhead), GFP-Me31B marking P-bodies in green (green arrowhead), and mRNAs colocalising with P-bodies in white (white arrowhead). All images are maximum projections of 7 Z slices of a confocal image. Scale bar: 5 μm. (B) Analysis of P-body sizes reveals an average radius of 0.2 μm. (Ci) Analysis of the correlation between Me31B binding from 2–3 hour embryos and (Cii) 3–4 hour embryos with model half-lives. A significant negative correlation (r = −0.16, p = 0.014) is found at 2–3 hours but no significant relationship is found at 3–4 hours (r = −0.07, p = 0.30). Underlying data can be found in S1 Data. (TIFF) [file pbio.3001956.s016.tiff]

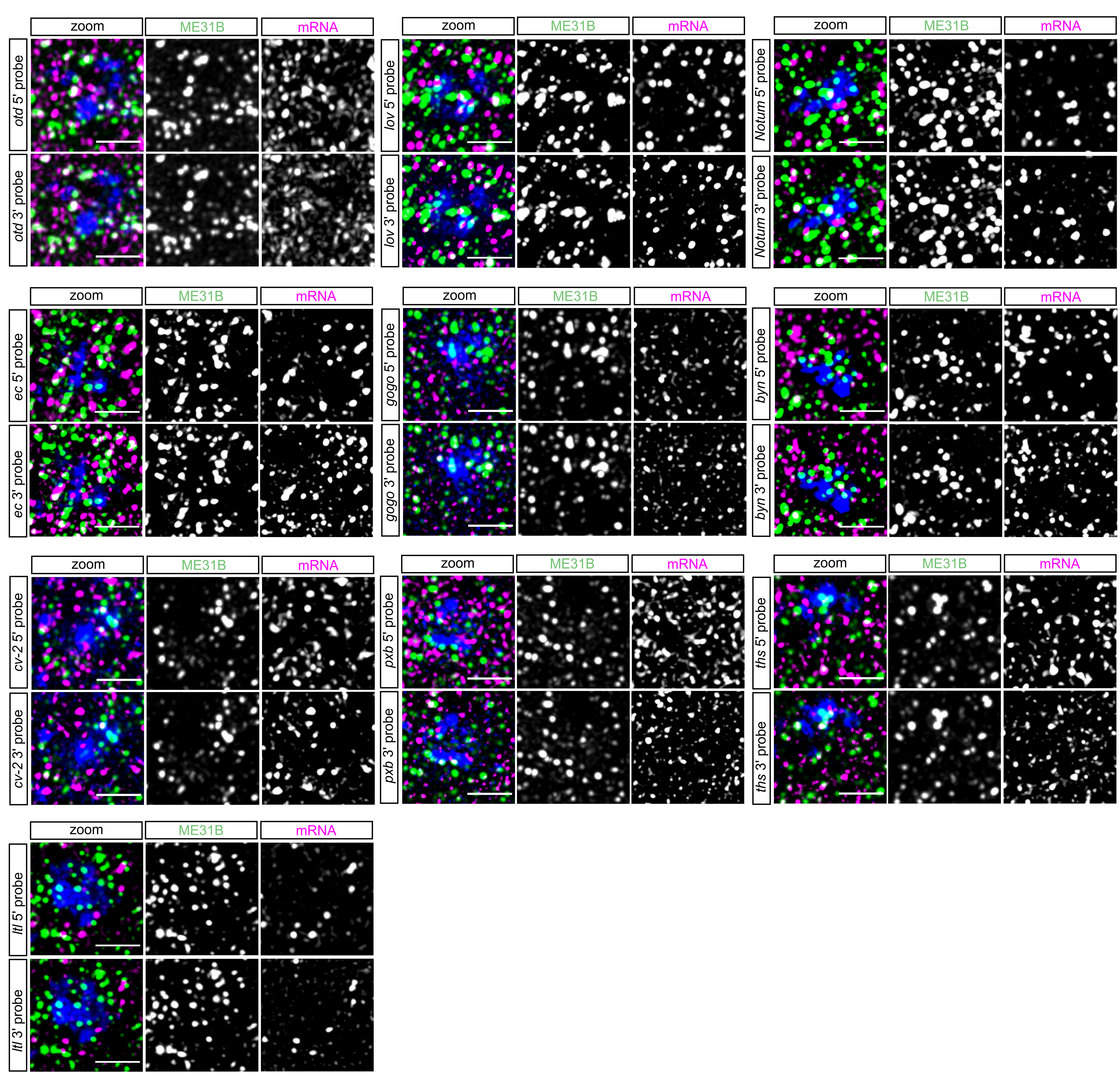

Supplement: S11 Fig — Confocal images of early nc14 Me31B-GFP embryos showing smFISH staining of the indicated test mRNAs with 5′ and 3′ probe sets. For each test mRNA, the same region of the embryo is shown with the 5′ and 3′ mRNA probe sets separately for clarity. mRNAs are shown in magenta, GFP-Me31B marking P-bodies in green, and DAPI labelling nuclei in blue. All images are maximum projections of 7 Z slices of a confocal image. Scale bar: 2 μm. (TIF) [file pbio.3001956.s017.tif]

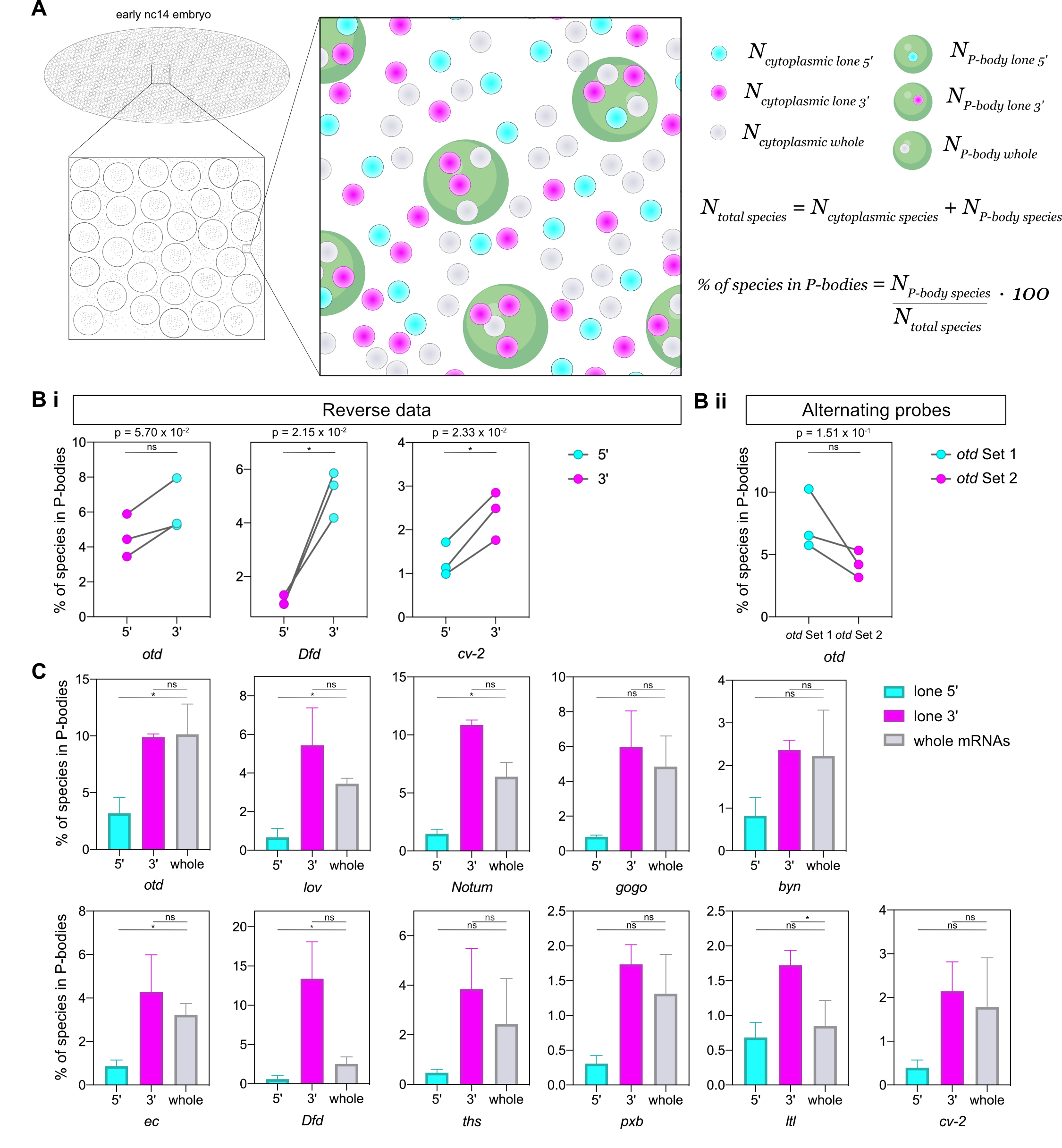

Supplement: S12 Fig — (A) Schematic demonstrating quantitation of the proportion of each species in P-bodies; 5′ and 3′ spots are detected and then paired (see Methods) to give 3 species—whole mRNAs (grey), lone 5′ ends (cyan), and lone 3′ ends (magenta). The number of each species that colocalises with P-bodies is divided by the total number of that species to give a percentage enrichment in P-bodies. (Bi) Quantification of the percentage of unpaired mRNA 5′ and 3′ ends in P-bodies relative to the total number of lone 5′ or 3′ ends in the switched probe fluorophore experiments for otd, Dfd, and cv-2 (see also Fig 6C). Paired t test was used to determine significance. (Bii) As in (Bi) but alternating otd probes were quantified. (C) Percentages of each species (whole, lone 5′ and lone 3′) in P-bodies across the test set of mRNAs. mRNAs are ordered by their half-life from the shortest (otd) to the longest (cv-2) half-life. One-way repeated measures ANOVA was used to determine significance with α = 0.05. Underlying data can be found in S1 Data. (TIFF) [file pbio.3001956.s018.tiff]
