# Supplementary material for: Combined modelling of mRNA decay dynamics and single-molecule imaging in the Drosophila embryo uncovers a role for P-bodies in 5′ to 3′ degradation
Source: PLoS Biol. 2023 Jan 17;21(1):e3001956. doi: 10.1371/journal.pbio.3001956 (PMC9882958; doi:10.1371/journal.pbio.3001956)
Supplement: Attachment — Submitted filename: Response to reviewers.docx [file pbio.3001956.s019.docx]

*Rev. 1:*

*In this manuscript, the authors derive mRNA half-life data from select mRNAs in early Drosophila embryogenesis taking advantage of the large changes in gene expression that occur when embryos switch to zygotic transcription. They analyze their data and correlate them with existing ribosome profiling results and other mRNA features. They conclude that mRNA half-life does not depend on translation efficiency but short-lived mRNAs tend to be more strongly associated with cytoplasmic P-bodies, membraneless organelles previously implicated in mRNA storage but also in mRNA decay. Overall, this is an interesting manuscript that should be of relevance to a broad readership interested in the post-transcriptional regulation of gene expression. However, there are also some significant weaknesses that need to be addressed prior to publication.*

*1. The main experimental weakness is the small number of mRNAs that are examined by smFISH in Figures 4 and 5. I am aware that these are likely technically involved experiments but these data sets need to be expanded in order to make them robust and meaningful. Currently, there is a large bias towards long-lived mRNAs and the authors need to add 2-3 additional short and medium half-life mRNAs each to confirm the current trends.*

We have now analysed five additional mRNAs so that each half-life category contains four transcripts. The results with these new mRNAs have been added into the compaction analysis and the P-body colocalisation experiments (Figures 4-6 and Figures S8-12). These new data confirm the trends previously described, strengthening the conclusions. Including additional mRNAs into the 3 stability categories also allowed us to present the data as categories and perform comparisons between them, as we have with our previous analysis, both for the compaction and P-body colocalisation analysis. We believe this is an improvement on the previous version where the mRNAs were presented and assessed by the order of their half-lives, as the uneven spacing between the genes could make the plots misleading. We thank the reviewers for this suggestion which has much improved these datasets.

*2. Is there a bias towards 3' end sequences in short-lived mRNAs in their global sequencing reads as their P-body association data would suggest?*

No, but we do not think we would expect a 3’ end bias as firstly only a small proportion of the mRNA is likely to be in P-bodies at any one time. Secondly, we now provide evidence that there are many 5’ and 3’ end mRNA fragments (Figure S9A and S9C and the related text in the paragraph starting on line 377). As we detect these in approximately equal amounts, we do not expect a general bias in one end of the mRNA.

*3. The writing of the manuscript needs to be improved:*

*a. The abstract overstates the conclusions and it should be clearly mentioned that the half-lives are derived from changes in intronic reads. It is also not correct that they can determine half-lives 'globally'. In addition, it must be clear from the abstract that their conclusions are based on correlations (or lack thereof).*

We have made all of these text changes in the abstract, and been more cautious with the interpretations throughout.

*b. There is no need to (overly) negatively discuss other approaches to measure half-lives such as metabolic labeling. Yes, the labeling times etc can introduce errors in metabolic approaches but it has been a powerful method to determine decay rates on a global scale in various systems. Technical limitations in the embryo fully together with the non-invasive nature justify their approach (but it also has limits and will not be generally applicable, which should be discussed).*

We have removed the description of the disadvantages of the other methods from the Introduction and now only mention them briefly in the Discussion when discussing the pros and cons of the available methods. With this, we also expand on the disadvantages of our approach.

*c. P13. The authors wanted to test "whether mRNA localisation influences stability". Again, the experiments allow them to correlate only and maybe stability influences localization instead…*

We have altered the text to make it clear we are investigating correlations.

*Rev. 2:*

*In this manuscript, the authors study the degradation of mRNAs during Drosophila embryogenesis. By staging embryos beginning at nuclear cycle 11 before zygotic transcription is turned on, single embryos were collected at intervals through nc11-14 and ending at cephalic furrow formation. RNA was isolated from these time points and intron and exon reads were used to measure changes in RNA levels during development. Since zygotic transcription is activated during their time course, they are able to identify transcriptions with intronic signals to specifically isolate transcripts whose dynamics can be followed from those mRNAs that are also maternally deposited. This data was then modelled using a Gaussian process to estimate transcript half-lives from the time-dependent changes in intronic and exonic reads. This allowed them to measure the stabilities of 279 transcripts (187 genes) and the differing intronic and exonic expression patterns were clustered that revealed that
transcripts with similar intronic dynamics displayed varying exonic dynamics suggesting that this resulted from differing mRNA half-lives. To determine if translation might influence mRNA stability, smFISH targeted to the 5' and 3' ends of transcripts was used to measure the compaction of mRNAs, which has previously been shown to correlate with active translation (elongated mRNAs are translating, while compact mRNAs are inactive). The authors further characterized the position of the 5' and 3' ends with respect to P-bodies and determined that unpaired 3' ends were enriched in P-bodies.*

*In general, I think the findings of this paper are interesting and could be an important contribution to the field, but there are some technical questions that should be addressed prior to publication*

*1. It was not clear to me how pre-mRNA transcript numbers were determined from their sequencing data. How are they accounting for the differences in length of introns and exons since a single intron could give rise to many reads?*

We have normalised the counts by the intron lengths so that the S parameter has comparable meaning for each mRNA. This is now mentioned in the main text and described in the Methods.

*Are the intronic reads evenly distributed?*

The intronic reads are evenly distributed and we have now included plots of the intronic (and exonic) read coverage across all time points in Figure S2A.

*Are they assuming all introns are co-transcriptionally spliced and then rapidly degraded?*

Yes, we believe these are reasonable assumptions as recent NET-seq data suggests that 95% of introns are co-transcriptionally spliced in the early embryo [1]. In addition, analysis of splicing by metabolic labelling in S2 cells found that the median intron half-life was only 2 minutes [2]. We have added this information to the text.

*I find it confusing that pre-mRNA levels are always higher than mature mRNA levels in Figure 2Di and Dii and believe this requires some clarification*

The pre-mRNA data were presented on the wrong scale in the previous version. We have corrected the figures to show comparable length-normalised units for the mRNA and pre-mRNA.

*2. In the modeling, they use a parameter S for splicing efficiency but I am not sure how this is defined. Do they assume every intron has the same splicing efficiency or all the introns of a particular transcript have the same efficiency? Can this be justified?*

If pre-mRNA is spliced into mRNA with a half-life of 2 mins then we would have a splicing rate S=0.35 in the model and we set that as the prior mean on the S parameter. However, we expect variation across genes and therefore the model is free to adapt S to fit the data for each gene. We assume that all the introns of a particular transcript have the same splicing efficiency, but that S can vary across transcripts. As described in the text, we consider this to be justified as there is evidence from *Drosophila* S2 cells that all introns within a mRNA tend to be spliced with similar rates [2].

*3. The authors claim that they are unable to perform intron and exon smFISH to determine mRNA half-lives because the embryo is not at steady state, but it is not clear to me how the measurements would be different from their sequencing data. Shouldn't it be possible to model it in a similar way?*

We could in principle model smFISH data in a similar way, but our aim with the smFISH approaches described was to validate the model with a completely independent approach. In addition, there are significant technical challenges associated with collecting smFISH data for biological replicates across the multiple time points required for the model. Firstly, we currently have no way of precisely ageing fixed embryos at or prior to nc13 that would impact on the number of the time points and accuracy between biological replicates, e.g. we could not generate an early and late nc13 time point as we did for the sequencing. Secondly, smFISH data tends to be noisy as there is greater technical variability in imaging methods and lower numbers of cells are investigated. This combined means that the numbers measured are more readily affected by noise than in bulk sequencing data, resulting in considerable biological variation in mRNA numbers between embryos [3]. Thirdly, the time required for the imaging/analysis means that smFISH is a low throughput method. For these reasons, we feel that it is outside the scope of this study to model smFISH time course data.

*4. In figure 4D, they show smFISH for the 5' and 3' ends of Dfd. They should also report what the total number of 5' spots, 3' spots, co-localization and detection efficiency. In the representative images, the detection efficiency looks to be low in Figure 4Dii.*

We are reporting typical detection efficiencies (Figure S9Bii), as well as the total number and relative proportions of 5’, 3’ and colocalised signals (Figure S9A and S9C). In Figure 4, the numbers are reported in the figure legend for the *lov* mRNA shown, and for Figure 6 we refer back to the numbers in Figure S9A and S9C so as to prevent including long lists of numbers in the figure legends. The referee is correct that we detect a relatively low proportion of dual signals, despite having a reasonable detection efficiency (~70% (Figure S9Bii), in the range reported in other smFISH studies). Our interpretation is that, for the mRNAs tested we are able to detect a population of degradation intermediates which lack either the 5’ or 3’ end. In support of this, the proportion of dual labelled mRNA species detected increases if we use alternating probes across the mRNA, rather than 5’ and 3’ probe sets (Figure S9E). We have explained these data in the Results section (the paragraph starting on line 377).

*It also surprises me that the FISH signal is so different in size and intensity in Figure 4Dii? Is this an artifact of deconvolution? The FISH signal looks more uniform in Sup Fig S8D and E though.*

This was in part an artifact associated with opting to only show a small number of confocal Z slices. We only show a few confocal Z slices so that it is easier to see single mRNAs (in the embryos there can be >100 copies of a mRNA/cell) as the density is lower when only a few slices are included. However, the choice of slices was poor so that only part of many signals were visible, giving an impression of different sizes and intensities. We have replaced the image in Figure 4D with one where we have chosen the slices to include more carefully. In addition, as *Drosophila* mRNAs are short, for some we are limited to 24 probes, resulting in more noise than when we use 30 probes. This is the case for *Dfd* (in the original Figure 4Dii), so the data has a lower signal to noise ratio. We now mention in the text that we consider using 24 probes to be close to the minimum number required for detection (line 381).

*5. In figure 5, the authors show the co-localization of different mRNAs with P-bodies. As Neu2 (unstable) and Dfd (stable) hare similar co-localization indexes, I find it somewhat overstated to claim that unstable mRNAs are enriched in P-bodies given the small number of mRNAs tested and the similarity stated above. There should at least be equal numbers of transcripts in each class for comparison.*

As described above (Reviewer 1, point 1), we have now added additional transcripts to the analysis so that each half-life category contains four transcripts. We have also made clear that the trends observed are for a set of test mRNAs only, and we have toned down the conclusions throughout.

*6. In Figure 6, the authors look at the co-localization of 5' and 3' ends of mRNAs with P-bodies to determine if decay is occurring in P-bodies. They need to also report the number of unpaired 5' and 3' ends in the cytosol as well and the detection efficiency in order to further support their observations. For example, if they are systematically under-detecting the 5'-ends, this would result in similar data, but with a very different conclusion.*

We now report the number of unpaired 5’ and 3’ ends in Figure S9A and the detection efficiency in Figure S9B (See Reviewer 2 point 4). As described (line 304), we see lower detection efficiency in the 670 channel. However we have reverse labelling controls for the majority of the mRNAs tested (Figure S9C) to further show that the data are not due to labelling/detection problems for one of the mRNA ends. We show that we are not systematically under-detecting the 5’ ends: we detect similar numbers of lone 5’ and 3’ ends for any given mRNA (Figure S9A and S9C), but the 3’ ends are more colocalised with P-bodies (Figure 6C).

*Minor comments.*

*1. I would suggest reformulating the title since modeling of mRNA stabilities from the RNA sequencing data did not reveal any about the location of mRNA decay and this was found by microscopy. I understand the desire to incorporate both aspects of the experiments performed in the title, but I find it to be confusing*

We have changed the title to indicate that we have used modelling and imaging to study mRNA degradation, but no longer highlight P-bodies.

*2. The timings of collecting the embryos is not clear to me. In the text, it states that embryos were collected at 5 min intervals during nc11-13 and 15 min intervals during nc13 and nc14, however, this time is very different from what is shown in Fig1A and Table 1. This might be obvious to a Drosophila specialist because an additional time delay is implied, but requires a little more explanation for non-experts*

We have clarified the timings in the main text (line 60) and indicated approximate timings of each nuclear cleavage cycle in Table 1 to help the reader.

*Rev. 3:*

*Review: Beadle, Love et al., "Modeling global mRNA dynamics during Drosophila embryogenesis reveals a relationship between mRNA degradation and P-bodies"*

*The authors present an exciting method to retrieve mRNA half-lives during Drosophila zygotic genome activation. For this they use a high-resolution RNA-seq time series, performed on carefully staged single Drosophila embryos. To estimate mRNA half-lives from these datasets, they employ a Gaussian process regression model, which jointly models pre-mRNA and mature mRNA. Having estimated mRNA half-life for ~300 zygotic transcripts, the authors then investigate the contribution of various steps of mRNA lifecycle to their stability. First, they use clustering and modeling to reveal that the time delay between peak pre-mRNA and mature mRNA levels could be used as a proxy to estimate mRNA stability. Next, they show that mRNA stability is not correlated with 3'UTR length, translation efficiency or codon optimality, but is moderately related to mRNA compaction. Finally, the authors assess the relationship between mRNA half-lives, their degradation and localization in p-bodies. For this, they focus on 7 zygotic transcripts, and employ smFISH labeling and quantitative imaging. For this small set of mRNAs, they show that unstable mRNAs (short half-lives) tend to locate in ME31B positive p-bodies, where they seem to be degraded.*

*The major strength of the manuscript lies in:*

*1) the exhaustive database of mRNA half-lives (very useful to the community), along with the methodology that could be easily transferred to other systems.*

*2) the description of correlations between mRNA half-lives and various post-transcriptional processes. I am less convinced by the part on p-bodies which primarily relies on a small set of examined mRNAs (see discussion below).*

*Apart from this last part, conclusions of the manuscript are well supported by the experimental data and bioinformatic analyses.*

*Conclusions of this paper are of immediate interest to many people in the field of gene expression and mRNA biogenesis in vivo. I recommend this paper to be published with minor corrections.*

*Here are my specific questions and recommendations (very minor):*

*#1.ME31B*

*I find the title too strong, as the major finding of the manuscript is not the link between mRNA degradation and p-bodies. Actually, a correlation between localization in p-bodies and mRNA half-life is shown for a handful of transcripts (=7). In the absence if experiments manipulating ME31B, this result remains correlative and should be interpreted as such.*

We have changed the title, which no longer mentions P-bodies.

*Similarly, I would nuance the conclusions in the results part, line 334. This is valid for 7 transcripts.*

We have nuanced the conclusions (now line 374) by saying that P-bodies in mRNAs may be sites of mRNA degradation for only a subset of mRNAs. We have also tried to be more careful about the conclusions elsewhere in the manuscript, e.g. we discuss (line 504) that even for the mRNAs we detect co-localised with P-bodies the presence of their 3’ ends outside P-bodies suggests that degradation is not limited to within P-bodies.

*To strengthen the observation that short-lived mRNAs tend to be localized within ME31B positive p-bodies, the authors could analyze ME31B RIP-seq datasets (if available for early embryos).*

We thank the reviewer for this suggestion. Me31B RIP-seq datasets from early embryos are available [4] and we show that there are significant negative correlations between Me31B binding and the model half-lives (Figure 5D; Figure S10C). We believe that these data strengthen our observation that short lived mRNAs tend to be more localised in P-bodies.

*#2. Modeling*

*The description of the Gaussian model is too vague in the main text. It is important to clearly state its assumptions and potential caveats in biological terms.*

We have improved the description of the Gaussian model in the main text including its assumptions and potential caveats (line 443).

*Would this methodology tend to over-estimate or under-estimate mRNA half-lives?*

We have provided additional simulations in the Supplementary Methods to address this question (Supplementary methods Figure S1). We have considered a range of different degradation parameters between 0.003 and 0.05 which corresponds to a range of half-lives between 231min and 14min. In all scenarios, the degradation parameter was estimated accurately by both maximum a posteriori estimation and MCMC sampling.

We added text about this in the Modelling section of the Methods (beginning line 720).

*Which temporal scale would be unreachable by this method?*

When the degradation is close to zero, the half-life is practically indistinguishable from infinity. We have provided additional simulations to investigate the limit of the temporal scale reachable by this model. In Supplementary Methods Figure S2, we demonstrate that half-life is estimated accurately up to a limit of 693.1min (D=0.001). However, once the degradation approaches zero, the degradation parameter becomes unidentifiable (i.e., it is estimated as small, but it cannot be inferred accurately anymore).

*Why choose the threshold 0.4 (line 139)? What would we gain/lose by being more/less stringent?*

When the data are uncorrelated, e.g. because technical or biological noise dominates the signal, it is technically hard to fit the model and the parameter estimation will often fail. We found 0.4 to be a mild correlation threshold where we keep all interesting examples with related dynamics while excluding the genes in which pre-mRNA and mRNA are unrelated. This saves computational time as we do not fit the model on the examples where the model would not have a good fit in any case. This justification has been added to the text (line 154).

We used a strict filter for selecting zygotic transcripts (TPM < 0.5 at the first time point 95min). Above this, we may start to include transcripts with a maternal contribution. Maternal transcripts are cleared with a different half-life to zygotic transcripts in some cases and therefore our model is not suitable for modelling genes with a combined maternal and zygotic contribution in its current form. Although we could potentially lose some truly zygotic transcripts with variation at t=95, we need high quality data for modelling and therefore ensuring we only included purely zygotic transcripts, at the cost of losing some noisier examples, was preferable.

*Simulations might be useful to clarify these points.*

As described above, we have simulated five data sets with different degradation rates, and discuss the model fits in the Supplementary Methods. When carrying out rigorous testing of the model during the simulations we discovered a bug which introduced errors in the degradation rate estimates. This was not immediately evident since the overall trends observed remain similar. Correcting this mistake has improved the results so that half-life estimates are more accurate (as shown on simulated data) and the trends supporting our results are stronger than those reported previously. This improvement has resulted in some changes in half-lives from the previous estimates included in the original version of the manuscript including some mRNAs switching categories.

*In terms of modeling, what are the advantages of using a Gaussian compared to other models? This is briefly mentioned in the discussion (line 380) but could be elaborated on a bit more.*

There are two main advantages: 1) Gaussian processes (GPs) are non-parametric and thus can fit various dynamics of gene expression without explicit parametric assumptions, which can be limited (we already mentioned this in the Discussion); 2) Uncertainty quantification: GPs lead to a natural uncertainty quantification framework about the possible underlying non-parametric functions. Additionally, in our approach, we are also quantifying uncertainty about the parameters of interest using Bayesian inference (MCMC). Because GPs can be integrated over analytically, the Bayesian MCMC only has to be done for relatively few parameters making it computationally feasible. We now also mention the second point in the Discussion in lines 440.

#3. mRNA half-life dataset

*Given that intronless zygotic transcripts could not be studied with this method, could the author state the % of total zygotic transcripts Table S1 represents (line 147)?*

Although the number of transcripts we can detect in our dataset is high (18159 transcripts detected > 1 FPM) we applied very strict filtering (see Methods section RNA-seq data processing and data filtering) to obtain only dynamic and zygotic transcripts. By taking these two filters into account, the number of zygotic AND dynamic transcripts is only 593. We then also exclude transcripts without introns and are left with 505 transcripts. In Table S1 we are able to obtain half-lives for 263 out of the 505 of these which represents 52% of intronless, dynamic, zygotic transcripts but only a small proportion of the total zygotic transcripts expressed during early embryonic development (5094 transcripts: 5% modelled).

*How does intron size affect this pipeline and the output?*

The intronic expression level is divided by the total intron length for each gene and the exonic expression level is similarly normalised. The transcript expression is TPM normalised which also takes transcript length into account. We have verified that the sum of TPMs across transcripts matches the length-normalised exon counts, so that the units for intronic, exonic and transcript expression are all length-normalised and comparable. In terms of intron size, intronless transcripts cannot be modelled using this approach and for genes with small introns, the intronic signal is not strong enough to pass the dynamic filter required for modelling and would therefore also be excluded.

*Part of the method assumes that pre-mRNA profiles reflect transcription dynamics. Could the authors use the recently published NET-seq results in early staged embryos to back up this assumption?*

We thank the reviewers for this suggestion and have validated our intronic data using the NET-seq data from early 2-3 hr embryos. For our modelled genes we see a significant positive correlation (Figure S2B, Spearman’s correlation coefficient = 0.46, p = 5.1e-11) between our intronic reads at the nc13 and nc14 timepoints from our data and the NET-seq reads. We believe that given these measurements are from different techniques, spanning a relatively wide developmental time window, this correlation validates that our pre-mRNA counts are indicative of transcription in the early embryo.

*Minor points*

*-The quality of the images Figure 1, S1A and could be improved, as well as their sizes.*

We have improved the quality by increasing the size and/or resolution of Figure 1 and S1A and these have now been updated in the manuscript.

*-The number of 'pairs' should be indicated in the figure legends of smFISH data using 3' and 5' probes.*

These data has been added to the legends of Figures 4 and 6 and can be found in Figure S9A and S9C (see Reviewer 2 point 4).

*-Figure 3: I would define the notion of 'delay' within Figure 3.*

We have added a definition (line 233) and updated the schematic in Figure 3C.
